# Mechanisms behind the testing effect: an empirical investigation of retrieval practice in meaningful learning

**DOI:** 10.3389/fpsyg.2015.01054

**Published:** 2015-07-24

**Authors:** Tino Endres, Alexander Renkl

**Affiliations:** Department of Psychology, University of Freiburg, Freiburg, Germany

**Keywords:** testing effect, elaborative retrieval, meaningful learning, mental effort, retrieval practice

## Abstract

The testing effect—more learning by testing as compared to restudying—is a well-established finding. A typical testing procedure in the context of meaningful learning comprises a recall task after an initial study phase. Different theories refer to different mechanisms when explaining the positive effects of such recall tasks. In the context of learning from expository texts, we tested three mechanisms as suggested by a variety of prominent approaches: the elaborative-retrieval theory, the theory of transfer-appropriate processing, and the unspecific-goal perspective. We experimentally varied the type of testing task (short-answer task vs. free-recall task, both compared to a restudy task) in a within-subject design (*N* = 47 university students). We replicated the testing effect. We found no evidence for a transfer-appropriate processing effect or an unspecific-goal effect. The testing effect disappeared when statistically controlling for mental effort. Initially non-tested material was also fostered by testing (spreading activation effect). These findings indicate that testing helps learning when learners must invest substantial mental effort, as suggested by the elaborative retrieval theory. For educational purposes, testing tasks should be assigned that require the learners to invest substantial mental effort.

## Introduction

Many studies have confirmed that testing improves retention over a period of several days or weeks. In a typical arrangement analyzing the testing effect, learners first study some materials, for example, a text (learning phase). Afterward they either take a test or restudy the text again (intervention phase: initial testing). Learners improve more by taking tests than by restudying the previously learned material, in particular when delayed learning outcomes (e.g., 1 week after learning) are considered (assessment phase). This effect has been observed with different kinds of test problems and in different subjects (Rowland, 2014). Although the testing effect is well-established, there is ongoing debate about the factors under which learning by testing works best (e.g., different types of intervention tasks), and which mechanisms are responsible for substantial testing effects (Rowland, 2014).

Different theories refer to different mechanisms when explaining the testing effect. In this study, we tested three mechanisms that are potentially relevant in meaningful learning from expository texts. Meaningful learning in this context denotes that the learning contents possess a logical internal structure (in contrast to, e.g., word list, as used in other testing effect studies). The learners are expected to understand this internal structure, and the testing questions tap this understanding.

These mechanisms were suggested by different theoretical approaches: elaborative retrieval theory (e.g., Carpenter, 2009), transfer appropriate processing theory (e.g., Morris et al., 1977) and an unspecific-goal perspective (e.g., Paas and Kirschner, 2012).

### Testing Effect in Meaningful Learning: Different Theoretical Approaches

The first approach is the elaborative retrieval theory (Carpenter, 2009), which is based on associative memory theories (e.g., Anderson et al., 1997) and the desirable difficulty framework (e.g., Bjork and Bjork, 2011). This theory predicts two processes relevant to learning by testing. The first is the concept of spreading activation. According to this concept, working on a test task does not only strengthen previously existing retrieval cues. Retrieval also builds up new cues by spreading the activation used for recalling related contents that are close in semantic memory. Hence, searching for specific contents in associative memory networks activates these specific contents and other contents associated with it, even if the latter contents are not directly retrieved. Carpenter (2009) originally developed this approach analyzing word lists experiments. However, testing effect studies also found support for spreading activation in meaningful learning (retrieval induced facilitation). For example, Chan et al. (2006) found that not just initially tested materials are more accessible in a delayed assessment task. Closely linked topics can also be accessed better, as long as the topics have a high level of coherence. This spreading activation is responsible for the semantic elaboration of the primary recall concept.

The second important process in elaborative retrieval theory is the degree of semantic elaboration. The degree of semantic elaboration depends on the invested mental effort directed to elaboration. This aspect of retrieval is widely discussed and has attracted broad support (Rowland, 2014). Within this account, a challenging test task is assumed to lead to higher invested mental effort and accordingly to more activation, which strengthens the directly retrieved learning contents (Halamish and Bjork, 2011) and spreads the activation in semantic memory to related contents (Carpenter, 2009). Against this background, the degree of semantic elaboration can predict how easily contents are retrievable. Mental effort can thus be considered a marker for semantic elaboration. Concerning their instructional consequences: testing tasks should be used that require each learner to invest substantial mental effort. A more difficult task should lead to greater elaboration as long as it is successfully solvable.

The second approach is the transfer appropriate processing theory (Morris et al., 1977). Its explanation of the testing effect is based on the assumption that the positive findings on long-term memory are a matter of transfer. Transfer is fostered by the similarity of cognitive processes during learning tasks and transfer tasks. Advantages of testing vs. restudy exist because the cognitive processes during transfer tasks are more similar to those during initial testing, as compared to restudying (e.g., Thomas and McDaniel, 2007). In addition, the testing effect should be stronger if the types of problems (e.g., free-recall or short-answer) during initial testing and during transfer or an assessment test are identical, because the cognitive processes should likewise be more similar. Most of the studies confirming a transfer appropriate processing perspective compared recognition processes and recall processes (e.g., Johnson and Mayer, 2009). Similar patterns of results have been forthcoming by comparing meaningful retrieval processes analogous to the present study (free-recall problems or short-answer problems; Nungester and Duchastel, 1982). An instructional consequence of the transfer-appropriate processing approach would be to deploy testing tasks for learning that require largely identical cognitive processes as do typical tasks in a learning domain. A closer fit should lead to better final learning manifested as knowledge application.

The third approach is the unspecific-goal perspective. Usually, learners acquire deeper knowledge when they pursue more general goals during learning, in contrast to very specific goals, such as finding a certain numerical result or fact (e.g., Vollmeyer and Burns, 2002; Paas and Kirschner, 2012; Renkl et al., in press). Hence, this perspective suggests that more open problems have a special advantage (e.g., free-recall problems) in conjunction with initial testing as compared to more specific tasks. This pattern of results was also revealed in many studies examining the testing effect (e.g., McDaniel and Masson, 1985; Foos and Fisher, 1988; Kang et al., 2007). In all of these studies, question types that tended to be unspecific fostered learning better than the more specific question types. A similar pattern became apparent in the effect sizes in current meta-analytic research (Rowland, 2014), although the different effect sizes (free-recall problems *g* = 0.81, cued-recall problems *g* = 0.72) did not differ significantly. An instructional consequence of the unspecific-goal perspective is to assign unspecific testing tasks for learning purposes. An unspecific task such as free-recall problems should foster learning to a greater extent than a more specific one such as short-answer problems.

To compare these three approaches, we experimentally varied the type of intervention testing task (free recall task vs. short answer task in addition to a restudy task). We assessed subjective mental effort during learning (as an indicator for elaboration). Furthermore, we analyzed the learning outcomes by assigning different types of test problems during the assessment posttest (free-recall problems and short-answer problems). The three different approaches enable different predictions to be made regarding the finding patterns in posttest outcomes:

The *elaborative retrieval hypothesis* predicts that the mental effort invested during the intervention phase accounts for the learning outcomes in both types of assessment problems (i.e., free-recall and short-answer test). In addition, the spreading activation assumption predicts that initially non-tested items should also reveal a testing effect for both posttest problems.

The *transfer appropriate processing hypothesis* predicts that learning is best if the testing tasks in the intervention phase and in the assessment phase are of the same type. We expect an interaction between intervention task type and assessment task type concerning the posttest performance.

The *unspecific-goal hypothesis* predicts that learning is best if an open initial task (free-recall) is used in the intervention phase. Note that these predictions do not necessarily rule each other out. Rather, two or even all three mechanisms the different theoretical approaches propose might contribute to the final pattern of results.

## Materials and Methods

### Subjects

Forty-seven university students (age: *M* = 23.2, SD = 3.4) of different majors participated in the study. None of the participants had diagnosed dyslexia or similar learning difficulties. The whole experiment followed the rules set by the ethical guidelines of the German Psychological Society’s (DGPs; 2004, CIII). Participating subjects were given 10 Euros or course credit for participation. All subjects were aware of taking part in research. Before starting the experiment we informed each subject about the possibility of quitting the experiment with no repercussions out disadvantage at any time. All participants provided informed consent and allowed us to use their collected data anonymously for publications. All data was anonymously collected and analyzed.

### Design

We applied a within-subject design. The factor intervention phase consisted of three conditions: restudy condition, free-recall condition, and short-answer condition. The dependent variables in the assessment phase comprised the posttest performance on free-recall problems and on short-answer problems, as well as an overall posttest score. There was also a variation with respect to the presentation of the short-answer tasks: three items were used in intervention phase. All six were used in the assessment phase. Conditions were distributed over texts to control for non-expected text effects.

### Materials

We used three expository texts. Each text dealt with different psychological topics; the texts had already proven useful in a similar experiment (Avci, 2011). As they were written in English, we translated them into German. The texts had an average length of 1186 (text 1:1220; text 2: 1020; text 3: 1318). We checked all material in a pilot phase. In the pilot phase, we measured reading time, which did not differ significantly among texts, *F*(2,22) = 0.983, *p* = 0.390. Neither did we note any text differences with respect to understandability, emotional effects and interest as perceived by the students (all *p*s > 0.20).

We developed two kinds of tasks for each text: one free-recall task and six short-answer task (three tasks for initial testing in the intervention phase, and three tasks for the assessment phase). In the free-recall task, students had to write down the content of one text. The short-answer problems addressed a particular concept in each of the texts (e.g., “Why did Braver use clinical and non-clinical subjects? What consequences might the Mau-Mau hypotheses have in the future?”). In the previous test phase, both question types showed similar time-on-task in intervention phase (one open question compared to three short answer questions). We observed no difference between task types in how the students rated understandability, emotional effects, and interest (all *p*s > 0.20). A coding schema was developed for the posttest that applied to both posttest types. We rated each conceptual aspect individually. The answers were scored according to our coding system. Twenty percent of the questions were rated by a second rater, which revealed high interrater agreement on both question types (short-answer problems ICC = 0.97, free-recall problems ICC = 0.93).

To assess subjective mental effort, participants were asked after every task to rate the mental effort necessary to complete the task. According to Sweller et al. (2011) this measure can be regarded as a reliable indicator of mental effort. To assure comparability with other studies examining elaborative-retrieval theory, we also assessed subjective item difficulty. In this study, subjective item difficulty can be considered a control variable. Subjective mental effort and subjective item difficulty were assessed with items using a scroll-bar (0% = low–100% = high).

### Procedure

The experimental sessions were computer-based. We kept time-on-task constant in every part of the experiment. The extent of the individual answers was controlled by a color-changing word counter placed right under the description field, indicating to participants how many alphabetic letters they had already written. This counter changed color from red to green when the required number of letters had been reached and returned to red when too many letters had been written (short-answer: 50–150; free recall 300–900). This procedure should help to get similar answer length in the different conditions, without triggering participants’ reactance by too intrusive procedures (e.g., enforcing answer of a particular length).

**FIGURE 1:**
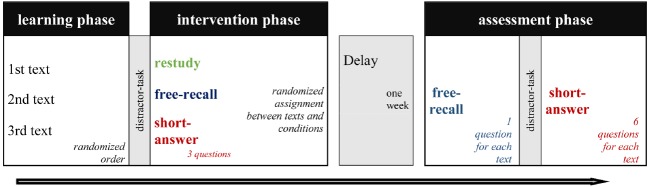
**Overview of the experimental procedure**.

In the experiment, we used an arrangement typical of testing-effect studies consisting of a learning phase, intervention phase, and assessment phase. To combine different conditions within one participant, each participant studied three different expository texts (see Figure 1).

There were two experimental sessions 1 week apart. In the first session, each participant read three expository texts. A following word-based distractor task (a guessing game called hangman) was used to avoid short-term memory use. Afterward, participants were tested in a free-recall format, short-answer format, or restudied one text (intervention phase). In each condition, participants read one of the three texts. The texts and conditions were randomized in order and combination (i.e., VP1: text 1 free-recall, text 2 short-answer, text 3 restudy VP2: text 2 restudy, text 3 free-recall, text 1 short-answer; etc.). In that way, we controlled for potential text and sequence effects (which we did not necessarily anticipate). Participants were asked to rate their mental effort and subjective difficulty after each task.

In the second session, the participants worked on both types of tasks (free-recall and short-answer) in all three texts, and each started with the free-recall format. A subsequent distractor task (hangman game) prevented short-term memory use. Finally, all participants answered all six short-answer problems (three initial tested and three initially non-tested items).

## Results

### Pre-analyses

Mental effort different substantially among the participants (see Table 1). However, there were no differences between conditions [*F*(1,92) = 0.93, *p* = 0.399]. Subjective difficulty correlated closely with mental effort (free-recall, *r* = 0.903, *p* < 0.001; short-answer problems, *r* = 0.720, *p* < 0.001).

**TABLE 1:** **Descriptive statistics of mental effort**.

**Mental effort**	**Minimum**	**Maximum**	***M***	**SD**
Restudy	0	90	42.17	25.71
Free-recall	5	89	47.40	25.35
Short-answer	0	100	49.30	27.39

Although we had controlled for perceived text difficulty in the previous test phase, the analysis of text-specific outcomes revealed significant differences between texts; for free-recall, *F*(2,92) = 4.07, *p* = 0.020, ${\text{η}}_{p}^{2}$ = 0.08; for short-answer, *F*(2,92) = 17.70, *p* < 0.001, ${\text{η}}_{p}^{2}$ = 0.28. We therefore used z-transformation of text-specific outcomes to control for different perceived text difficulties.

All tasks had a possible maximum of 15 points according to our coding system (six short-answer questions or one open-answer question). After rating all items we applied a range from 0 to 14 points in text 1, 0 to 13 points in text 2 and 0 to 13 points in text 3. Item difficulty differed slightly among texts (text 1: 6,3_*short–answer*_ 5,4_*open–answer*_ text 2: 6,5 _*short–answer*_ 6,24_*open–answer*_ text 3: 4,9_*short–answer*_ 5,34_*open–answer*_). This slight difference was already adjusted by applying the z-transformation of text-specific outcomes.

### Main Analysis

First, we analyzed the posttest outcomes (see Table 2). Analysis of variance for repeated measurements for both assessment problems revealed a significant effect of condition on overall posttest scores *F*(2,92) = 8.58, *p* = 0.001, ${\text{η}}_{p}^{2}$ = 0.16. The contrast between restudy and the two test formats attained statistical significance [*F*(1,46) = 16.51, *p* < 0.001, ${\text{η}}_{p}^{2}$ = 0.264], indicating a testing effect. Examining the two different types of posttest tasks, we detected the same pattern of results: significant effect of condition: free-recall posttest *F*(2,92) = 8.58, *p* < 0.001, ${\text{η}}_{p}^{2}$ = 0.157; short-answer posttest, *F*(2,92) = 5.58, *p* = 0.005, ${\text{η}}_{p}^{2}$ = 0.11. The contrasts between restudy of the two initial test formats were significant for each type of assessment problem [free-recall posttest, *F*(1,46) = 16.06, *p* < 0.001, ${\text{η}}_{p}^{2}$ = 0.259; short-answer posttest, *F*(1,46) = 9.57, *p* = 0.003, ${\text{η}}_{p}^{2}$ = 0.172], indicating a testing effect. The effect sizes of the testing effect in the short-answer posttest were as follows: open answer *d*_*rm*_ = 0.44, short answer *d*_*rm*_ = 1.28; open answer posttest: open answer *d*_*rm*_ = 1.26, short answer *d*_*rm*_ = 1.72. These effect sizes are somewhat higher than the usual size of testing effects [see the recent meta-analytic review by Rowland (2014)]. These effect sizes can be regarded as a consequence of the high statistical power of our within-subject design and the focus on meaningful learning.

**TABLE 2:** **Descriptive statistics of posttest outcomes**.

**Assessment problem**	**Intervention task**	***N***	**Minimum**	**Maximum**	***M***	**SD**
	Restudy	47	–3.08	1.59	–0.24	0.91
Short-answer	Free-recall	47	–2.11	2.10	–0.04	1.08
	Short answer	47	–1.69	2.55	0.29	0.93
	Restudy	47	–1.84	1.83	–0.40	0.79
Free recall	Free-recall	47	–1.97	2.90	0.13	1.08
	Short answer	47	–1.94	2.79	0.27	0.97

We observed no interaction between the intervention task type and assessment task type with respect to posttest performance, *F*(1,46) = 1.02, *p* = 0.317, ${\text{η}}_{p}^{2}$ = 0.02, a finding that fails to confirm the transfer-appropriate processing hypothesis. We found no significant differences between initial test types (i.e., free call vs. short answer) with respect to the posttest performance [overall *F*(1,46) = 2.54, *p* = 0.118, ${\text{η}}_{p}^{2}$ = 0.052; free-recall performance *F*(1,46) = 0.76, *p* = 0.387, ${\text{η}}_{p}^{2}$ = 0.02; short answer performance *F*(1,46) = 3.35, *p* = 0.074, ${\text{η}}_{p}^{2}$ = 0.068], results that do not confirm the unspecific-goal hypothesis (which would have predicted an advantage for free recall during the intervention phase).

When statistically controlling for mental effort, the posttest differences in the conditions disappeared, which was true for the overall posttest score [*F*(2,92) = 0.00, *p* = 1, ${\text{η}}_{p}^{2}$ = 0.000], free-recall performance [*F*(2,92) = 0.00, *p* = 1, ${\text{η}}_{p}^{2}$ = 0.000], as well as short-answer performance [*F*(2,92) = 0.00, *p* = 1, ${\text{η}}_{p}^{2}$ = 0.000]. These findings indicate that learning outcomes can be accounted by mental effort, as predicted by the elaborative retrieval hypothesis.

We further analyzed the spreading activation assumption as included in the elaborative retrieval hypothesis. For that purpose, we tested performance differences with respect to the initially non-tested items (see Table 3). For the initially non-tested items, the short-answer testing conditions significantly outperformed the restudy condition, *F*(1,46) = 4.44, *p* = 0.041, ${\text{η}}_{p}^{2}$ = 0.088, thus confirming the spreading activation assumption.

**TABLE 3:** **Descriptive statistics of posttest outcomes of initially non-tested items**.

**Assessment problem**	**Intervention task**	***N***	**Minimum**	**Maximum**	***M***	**SD**
Short-answer	Restudy	47	–1.59	1.81	–0.21	0.93
	Short answer	47	–1.59	2.11	0.12	0.97

For exploratory reasons we analyzed the differences between the short-answer condition and free-recall condition with regard to the performance on non-tested items. There was no difference in the non-tested items, *t*(46) = 0.226, *p* = 0.822. Specific effects of testing problems only became apparent in the comparison of initially-tested and non-tested questions. The posttest differences between initially-tested items and initially non-tested items differed significantly within the short-answer condition [*t*(46) = 1.705, *p*_*one–tailed*_ = 0.048], but did not differ significantly in the free-recall condition [*t*(46) = 0.087, *p*_*one–tailed*_ = 0.465].

## Discussion

### Review of Hypothesis

We replicated the testing effect, although we found no evidence for the transfer appropriate processing hypothesis or unspecific-goal hypothesis. Subjective mental effort accounted for the differences between the conditions. Greater accessibility of initially non-tested items showed a spreading activation effect. This result pattern supports the elaborative retrieval hypothesis.

Our results underline the important role of mental effort when trying to exploit the testing effect in classrooms, as suggested by the elaborative retrieval hypothesis. Our findings on the initially non-tested items also support the elaborative retrieval hypothesis. The posttest scores of the initially non-tested items were raised by initial short-answer testing. Non-directly retrieved material that was closely associated was retrieved more easily. We interpret this as an indirect activation via effortful retrieval in short-answer problems leading to spreading activation. Overall, our findings on mental effort and non-tested items support the elaborative retrieval hypothesis, including the interpretation of mental effort as an indicator of semantic elaboration.

With respect to the transfer appropriate processing theory: we did not observe that learning was best when the initial testing and posttest items are the same, a finding that suggests a rejection of the transfer appropriate processing hypothesis. However, from the transfer-appropriate perspective, there might be another explanation for our finding. The degree of overlap between these cognitive processes might be too high to elicit a difference between cued-recall and free-recall in posttest scores. Transfer-appropriate processing might only apply when more divergent cognitive processes are crucial, such as those involved in recognition and free-recall. An argument to counter this alternative explanation arises from the lack of differences between the more diverse conditions as far as cognitive processes are concerned. There was no difference between restudy and any testing condition (short-answer problems and free-recall) after controlling for mental effort. There would have been a difference beyond mental effort at least between the testing conditions and restudy if cognitive processes were the key factor in explaining the testing effect. Hence, the degree of overlap seems unlikely as a valid explanation for the present case. All in all, the transfer-appropriate processing approach failed to explain the patterns of results in our experiment.

We did not observe evidence for the unspecific-goal hypothesis. Rather, our study results reveal a descriptive advantage of the more specific answer format (short-answer problems). There are two possible explanations for previous findings showing generally positive effects of free-recall. In line with our line of argumentation, the usual effects might be attributable to the greater difficulty of unspecific questions. The questions in the present study were moderately difficult, and both questions types were of similar difficulty. This comparable difficulty might explain why free-recall tasks were not superior in this study.

Another explanation for the divergent findings in the present study could be the use of conceptual questions only. Previous findings of generally positive effects may lie in the comparison of (relatively free) conceptual questions and (narrower) fact-oriented questions (cf. Vollmeyer and Burns, 2002).

### Instructional Consequences

One of the instructional consequences of the present findings is that task format does not matter that much in meaningful text learning. Our results suggest that testing tasks should be used that require learners to invest substantial mental effort. A more difficult task leads to more elaboration as long as it can be solved (more or less) successfully. This study’s particular merit lies in our focus on this factor. The mental effort rating was independent of the specific posttest task, highlighting the important role that the subjectivity of mental effort ratings plays apart from format. Another advantage of this rating is its easy application in this context. This easy assessment makes it possible to use mental effort in a variety of instructional situations.

Instructors should use these findings to help individualize testing tasks involved in retrieval while keeping the mental effort that has to be invested in mind. Advanced learners should be assigned more difficult tasks than beginners. In classrooms, this differentiation could be implemented with easier tasks in beginner classes and tasks of greater difficulty for advanced classes. In digital learning environments, algorithms could be used to individualize learning. Similar to other adaptations in cognitive tutors (Schwonke et al., 2011), sophisticated adjustments to previous mental effort ratings could be made. Related research was done by Salden et al. (2004) using mental effort and procedural tasks. Digital learning environments enable us to adapt questions for different learners; “tailoring” tests to the individual mental effort would optimize their learning processes.

### Future Studies

An interesting aspect deserving further research is the potential of format-specific effects. It seems likely that different testing formats have special learning advantages. The benefit of free-recall seems to be that everyone should be able to answer some aspects of a question. A too difficult task that leads to no retrieval at all is practically impossible with free-recall testing. Specific questions such as short-answer problems can be too difficult (i.e., no retrieval is possible). It also seems likely that spreading activation depends on specific task formats. Unspecific answers such as free-recall should lead to the broader but less intense activation of related information. A more specific task such as short-answer problems should intensify activation, especially in the tested topic and closely associated concepts. An indication of such item-specific effects is found in our pattern of results in the initially non-tested items. There was no significant difference between exposed and initially non-tested items in free-recall problems, but there was in the short-answer problems. This may be an indication of deeper but more limited activation in conjunction with short-answer problems. The last specific effect of format could be due to metacognitive factors. In this context, a more specific question should lead to higher metacognitive awareness of one’s own knowledge gaps, because these gaps are more readily revealed by the specific-question type. An illusion of understanding is less probable. Further studies could investigate format-specific effects on metacognition.

## Conclusion

This study supports the elaborative retrieval theory in the context of meaningful learning. Both hypothesized aspects of this theory (i.e., spreading activation and mental effort as degree of elaboration) have been confirmed. A potential instructional consequence is knowing that testing facilitates learning particularly when learners invest substantial mental effort. To fully exploit the testing effect, testing tasks should be assigned that require learners to invest substantial mental effort, as long as the tasks can still be solved (more or less) successfully.

### Conflict of Interest Statement

The authors declare that the research was conducted in the absence of any commercial or financial relationships that could be construed as a potential conflict of interest.

## References

[B1] Anderson J. Matessa M. Lebiere C. (1997). ACT-R: a theory of higher level cognition and its relation to visual attention. Hum. Comput. Interact 12, 439–462. 10.1207/s15327051hci1204_5

[B2] Avci G. (2011). Transfer of the Testing Effect: Just How Powerful is it? Ph.D. thesis, Rice University, Houston.

[B3] Bjork E. L. Bjork R. A. (2011). “Making things hard on yourself, but in a good way: creating desirable difficulties to enhance learning,” in Psychology and the Real World: Essays Illustrating Fundamental Contributions to Society, eds Gernsbacher M. A. Pew R. W. Hough L. M. Pomerantz J. R. (New York, NY: Worth Publishers), 56–64.

[B4] Carpenter S. K. (2009). Cue strength as a moderator of the testing effect: the benefits of elaborative retrieval. J. Exp. Psychol. Learn. Mem. Cogn. 35, 1563–1569. 10.1037/a0017021 19857026

[B5] Chan J. C. K. McDermott K. B. Roediger H. L. (2006). Retrieval-induced facilitation: initially nontested material can benefit from prior testing of related material. J. Exp. Psychol. Gen. 135, 553–571. 10.1037/0096-3445.135.4.553 17087573

[B6] Foos P. Fisher R. (1988). Using tests as learning opportunities. J. Educ. Psychol. 80, 179–183. 10.1037//0022-0663.80.2.179

[B7] Halamish V. Bjork R. A. (2011). When does testing enhance retention? A distribution-based interpretation of retrieval as a memory modifier. J. Exp. Psychol. Learn. Mem. Cogn. 37, 801–812. 10.1037/a0023219 21480751

[B8] Johnson C. I. Mayer R. E. (2009). A testing effect with multimedia learning. J. Educ. Psychol. 101, 621–629. 10.1037/a0015183

[B9] Kang S. H. K. McDermott K. B. Roediger H. L. (2007). Test format and corrective feedback modify the effect of testing on long-term retention. Eur. J. Cogn. Psychol. 19, 528–558. 10.1080/09541440601056620

[B10] McDaniel M. A. Masson M. E. (1985). Altering memory representations through retrieval. J. Exp. Psychol. Learn. Mem. Cogn. 11, 371–385. 10.1037/0278-7393.11.2.371

[B11] Morris C. D. Bransford J. D. Franks J. J. (1977). Levels of processing versus transfer appropriate processing. J. Verbal Learning Verbal Behav. 16, 519–533. 10.1016/S0022-5371(77)80016-9

[B12] Nungester R. J. Duchastel P. C. (1982). Testing versus review: effects on retention. J. Educ. Psychol. 74, 18–22. 10.1037//0022-0663.74.1.18

[B13] Paas F. Kirschner F. (2012). “The goal-free effect,” in Encyclopedia of the Sciences of Learning, ed. Seel N. M. (Netherlands: Springer), 1375–1377.

[B14] Renkl A. Skuballa I. T. Schwonke R. Harr N. Leber J. (in press). The effects of rapid assessments and adaptive restudy prompts in multimedia learning. Educational Technology & Society.

[B15] Rowland C. A. (2014). The effect of testing versus restudy on retention: a meta-analytic review of the testing effect. Psychol. Bull. 140, 1432–1463. 10.1037/a0037559 25150680

[B16] Salden R. J. C. M. Paas F. Broers N. J. van Merrienboer J. J. G. (2004). Mental effort and performance as determinants for the dynamic selection of learning tasks in air traffic control training. Instr. Sci. 32, 153–172. 10.1023/B:TRUC.0000021814.03996.ff

[B17] Schwonke R. Renkl A. Salden R. Aleven V. (2011). Effects of different ratios of worked solution steps and problem solving opportunities on cognitive load and learning outcomes. Comput. Human Behav. 27, 58–62. 10.1016/j.chb.2010.03.037

[B18] Sweller J. Ayres P. Kalyuga S. (2011). Cognitive Load Theory. Heidelberg: Springer.

[B19] Thomas A. K. McDaniel M. A. (2007). The negative cascade of incongruent generative study-test processing in memory and metacomprehension. Mem. Cogn. 35, 668–678. 10.3758/BF03193305 17848025

[B20] Vollmeyer R. Burns B. D. (2002). Goal specificity and learning with a hypermedia program. Exp. Psychol. 49, 98–108. 10.1027//1618-3169.49.2.98 12053536

